# Challenges in Managing External Dental Fistula in an Immunocompromised Elderly Patient: A Case Report

**DOI:** 10.7759/cureus.69742

**Published:** 2024-09-19

**Authors:** Chihiro Uda, Ryuichi Ohta, Takashi Koike, Chiaki Sano

**Affiliations:** 1 Family Medicine, Fuchu Hospital, Osaka, JPN; 2 Community Care, Unnan City Hospital, Unnan, JPN; 3 Oral and Maxillofacial Surgery, Unnan City Hospital, Unnan, JPN; 4 Community Medicine Management, Shimane University Faculty of Medicine, Izumo, JPN

**Keywords:** chronic inflammation, external dental fistula, idiopathic thrombocytopenic purpura, immunocompromised host, maxillary diseases, multidisciplinary care

## Abstract

This case report discusses a 69-year-old immunocompromised woman who presented with dyspnea and lower back pain, later diagnosed with an external dental fistula following the observation of a mass and pus discharge on her right cheek. The patient’s medical history included idiopathic thrombocytopenic purpura (ITP) and long-term use of immunosuppressive medications, complicating her condition. The fistula was linked to chronic inflammation that had progressed to maxillary bone destruction, requiring both antibiotic therapy and oral surgical intervention. Despite the initial challenge of diagnosing this rare and atypical presentation in elderly, immunosuppressed patients, comprehensive treatment improved her inflammatory markers and overall condition. This case highlights the importance of early detection and multidisciplinary management in preventing severe complications in patients with complex medical histories. It also emphasizes the need for heightened awareness of dental infections in patients with systemic conditions, mainly when presenting atypical symptoms.

## Introduction

An external dental fistula is a dental condition characterized by the formation of a fistulous tract, which serves as an excretory pathway for chronic purulent inflammation originating in the root area due to an odontogenic infection. This tract perforates the jawbone, extends through the subcutaneous tissue, and ultimately reaches the external skin of the oral cavity, forming fistulas and granulomas [1]. Throughout this process, symptoms related to the affected tooth are rarely reported [2]. The reported causes of an external dental fistula include apical periodontitis, impacted teeth associated with psoriasis, sequestrum, and chronic purulent lesions [3]. Concerning the teeth involved in the fistula, it has been observed that in relatively younger patients under 50 years of age, the mandibular molar region, particularly the first mandibular molar, is often implicated [3].

In contrast, in patients over 50, the anterior teeth, especially the canines, tend to be involved [4]. Although this condition falls within the dental domain, due to the specificity of its symptoms, over 60% of patients initially seek medical care in departments such as dermatology, surgery, plastic surgery, internal medicine, and otorhinolaryngology [5]. Misdiagnosis and mistreatment, stemming from a lack of awareness of this condition, have resulted in cases where patients undergo repeated excisions, biopsies, and prolonged antibiotic therapy, subjecting them to unnecessary burdens [6].

In this report, we present the case of a woman in her 60s who was diagnosed with an external dental fistula and a subperiosteal abscess in the anterior maxillary region, presenting with swelling of the right cheek and difficulty opening her mouth. Incision and drainage were performed, followed by curettage treatment of the causative tooth, leading to improved symptoms and a return to independent activities of daily living (ADL). Through this case, we discuss how general physicians in regional hospitals can diagnose and treat external dental fistulas and their progression to subperiosteal abscesses in the anterior maxillary region.

## Case presentation

A 69-year-old woman presented to a rural community hospital with chief complaints of dyspnea and lower back pain. She had been hospitalized for idiopathic thrombocytopenic purpura (ITP) until five months prior. Following discharge, she remained symptom-free and stable for about a month but subsequently developed exertional dyspnea, mainly during activities like bathing, which limited her daily activities. Her symptoms progressively worsened, leading to reduced food intake, bilateral lower leg edema, and difficulty in movement for 4-5 days before visiting the community hospital. Her medical history included myocardial infarction, hypertension, dyslipidemia, osteoporosis, right septic arthritis, and ITP. Her current medications included prednisolone 10 mg, fluconazole 100 mg, magnesium oxide, fexofenadine 120 mg, eltrombopag 25 mg, minodronic acid 50 mg, acetaminophen 1500 mg, aspirin 100 mg, dapagliflozin 10 mg, bisoprolol 0.625 mg, rosuvastatin 10 mg, and ezetimibe 10 mg daily.

**Figure 1:**
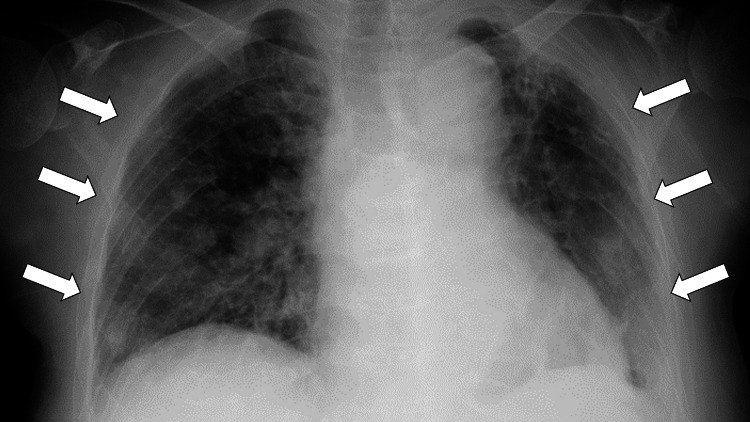
A chest X-ray demonstrating bilateral pulmonary congestion and pleural effusion

Upon her initial presentation, she was conscious and alert. Her vital signs were as follows: body temperature of 36.7°C, blood pressure of 128/76 mmHg, pulse rate of 72 beats per minute, and oxygen saturation of 98% on room air. Pitting edema was noted in both lower legs. The right side of her face was also slightly swollen and mildly tender. A cardiac examination revealed a third heart sound and a systolic murmur with peak intensity at the left sternal border in the second intercostal space. A chest X-ray demonstrated bilateral pulmonary congestion and pleural effusion (Figure 1).

Lumbar magnetic resonance imaging, conducted to evaluate her back pain, revealed an L1 vertebral fracture, a new vertebral fracture at the superior endplate of L3, and disc bulging at L4/5. Initial blood tests showed elevated C-reactive protein levels, but infection was not strongly suspected in the absence of fever at home (Table 1).

**Table 1:** Initial laboratory data of the patient CRP: C-reactive protein; Ig: immunoglobulin

Marker	Level	Reference
White blood cells	12.3	3.5-9.1 × 10^3^/μL
Neutrophils	86.5	44.0-72.0%
Lymphocytes	3.7	18.0-59.0%
Hemoglobin	10.6	11.3-15.2 g/dL
Hematocrit	33.8	33.4-44.9%
Mean corpuscular volume	85.6	79.0-100.0 fl
Platelets	11.7	13.0-36.9 × 10^4^/μL
Total protein	5.3	6.5-8.3 g/dL
Albumin	2.7	3.8-5.3 g/dL
Total bilirubin	0.6	0.2-1.2 mg/dL
Aspartate aminotransferase	21	8-38 IU/L
Alanine aminotransferase	16	4-43 IU/L
Lactate dehydrogenase	258	121-245 U/L
Blood urea nitrogen	27.8	8-20 mg/dL
Creatinine	1.00	0.40-1.10 mg/dL
Serum Na	145	135-150 mEq/L
Serum K	2.2	3.5-5.3 mEq/L
Serum Cl	101	98-110 mEq/L
CRP	6.53	<0.30 mg/dL
Urine test		
Leukocyte	Negative	Negative
Protein	Negative	Negative
Blood	Negative	Negative

Given the clinical suspicion of heart failure, intravenous furosemide 20 mg was administered. For the treatment of lower lumbar compression fractures, she was consulted by an orthopedic surgeon and managed conservatively. The lower leg edema improved the following day, and her dyspnea was alleviated, though it did not completely resolve.

**Figure 2:**
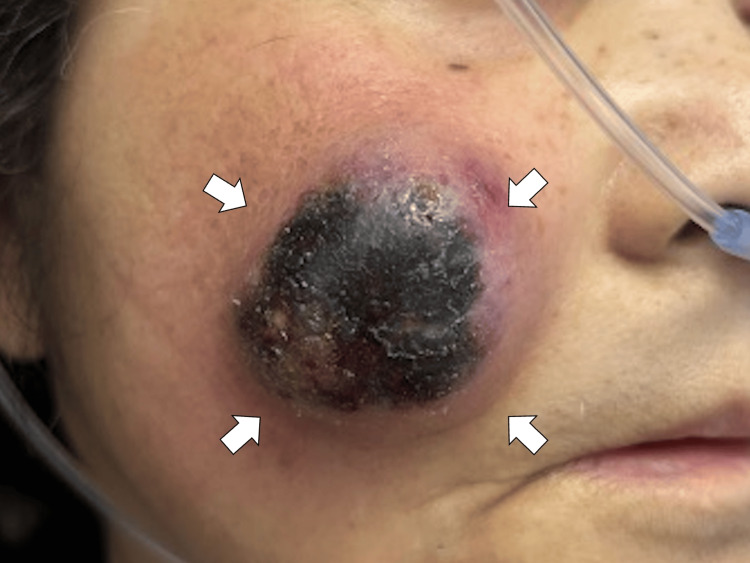
The swelling on her right cheek gradually enlarged with the surface black (white arrows)

On the second and third days of hospitalization, the swelling on her right cheek gradually enlarged, and the surface became necrotic (Figure 2).

**Figure 3:**
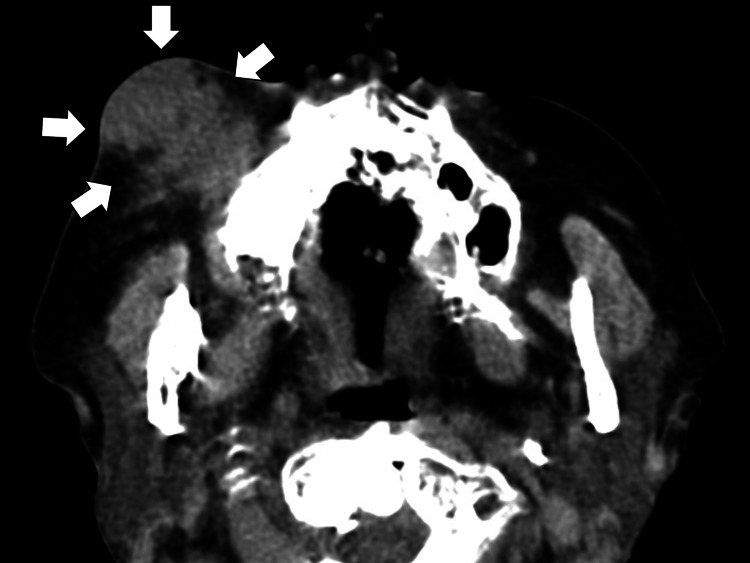
The facial CT revealing a suspected abscess anterior to the right maxillary sinus (white arrows)

A facial computed tomography (CT) scan was performed, revealing bone destruction in the maxillary region, particularly around the maxillary premolar, with a suspected abscess in the maxillary sinus (Figure 3).

**Figure 4:**
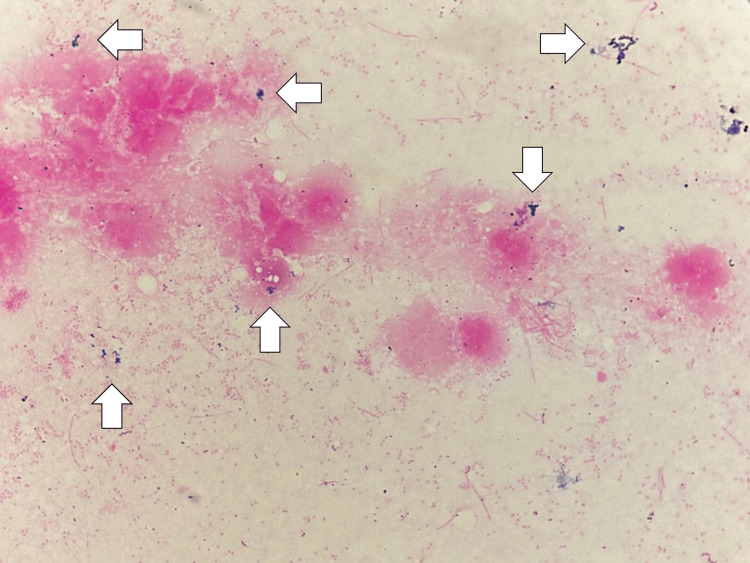
Gram stain of the pus of the abscess anterior to right maxillary sinus showing gram-positive coccus (white arrows)

A mass extending from the maxillary sinus to the cheek was also observed. On the fifth day of hospitalization, as the possibility of a skin tumor could not be ruled out, and with the mass swelling persisting on the right cheek along with pus discharge, she was diagnosed with an external dental fistula and maxillary osteitis, leading to an epi-maxillary abscess. Incision and drainage were performed, and she was started on ampicillin and sulbactam 4.5 g/day. Her dyspnea and heart failure could be triggered by systemic inflammation of the external dental fistula. Elevated CRP levels and white blood cell counts were consistent with the patient's systemic inflammatory response, likely driven by both the external dental fistula and possible cardiac complications. A Gram stain of the pus revealed gram-positive cocci (Figure 4).

The dermatology department initiated treatment with silver sulfadiazine cream on the same day. Subsequently, on the sixth day of hospitalization, the dental and oral surgery department performed curettage of the maxillary first premolar, which showed bone destruction. She said that she could not brush her teeth enough because of the bleeding tendency from ITP. The pus culture identified *Parvimonas micra*, which is sensitive to ampicillin and sulbactam. She was successfully treated with surgery and antibiotics without the progression to sepsis. We followed up on changes in symptoms, vital signs, and physical findings accompanied by the decreased CRP. Antibiotic therapy was continued until the 17th day of hospitalization, alleviating her dyspnea, fever, facial pain, and facial swelling. She was transferred to orthopedics for continued rehabilitation in preparation for the discharge to home.

## Discussion

In this case, a 69-year-old woman with immunodeficiency presented with dyspnea and lower back pain. She was diagnosed with an external dental fistula after the observation of a mass and pus discharge on her right cheek. Antibiotic treatment was administered, and oral surgical procedures were performed to address the maxillary bone destruction, improving inflammatory markers. This case highlights the need for comprehensive management considering the interrelationship of multi-organ complications, such as heart failure, fractures, and infections, which were progressing in general medicine [7]. Early detection and appropriate treatment of diseases, especially in older and immunocompromised states, are crucial in community hospitals [8]. It is also essential to consider how the patient’s overall condition was influenced by her history of ITP, past infections, and long-term use of prednisolone and antibiotics and how these factors impacted the treatment plan [9]. This case underscores the challenges of treating patients with complex medical histories and polypharmacy.

External dental fistula is a condition in which chronic purulent inflammation associated with odontogenic infection progresses, leading to the formation of fistulas and granulomas as the prolonged inflammation extends to the external skin of the oral cavity through the subcutaneous tissue [1,2]. A search in the Japan Medical Abstracts Society database revealed that 59.1% of reported cases of external dental fistula occurred in men, slightly more than in women [1,2,4]. However, this difference has narrowed recently due to improved oral hygiene awareness among men [10]. Regarding the location, 26.7% of cases involved maxillary teeth, while 73.3% involved mandibular teeth, with mandibular teeth being more commonly affected [10]. This is likely because when maxillary teeth are involved, the roots are closer to the maxillary sinus, increasing the likelihood of maxillary sinusitis [11]. Caries in maxillary teeth also tend to present with symptoms early, leading to earlier detection and treatment, thereby reducing the likelihood of progression to an external dental fistula [12]. However, the infection had progressed from the maxillary sinus to an external dental fistula in the present case. This could be attributed to a delayed inflammatory response due to immunosuppression and desensitization to pain caused by the patient’s medical history.

In elderly patients with immunosuppression, external dental fistulas may present with atypical clinical courses. Previous literature indicates that the prevalence of external dental fistulas is higher in individuals over 50 years old (64.8%) compared to those under 50 (35.2%) [1,4]. Furthermore, in those under 50, the condition is most commonly associated with molars (65.5%), while in those over 50, it is more frequently associated with anterior teeth, including canines (67.2%) [13]. This trend is likely due to the higher incidence of caries in the mandibular molars, especially the first molar, in younger individuals and the greater likelihood of caries in canines in older individuals [13]. The first molar is often implicated in younger patients because it is the earliest permanent tooth to erupt [1,4]. Its posterior position in the dental arch makes proper brushing difficult due to anatomical factors. In this case, the patient’s tendency toward bleeding due to ITP likely made maintaining dental hygiene challenging, and the resulting deterioration of oral hygiene in an immunosuppressed state may have contributed to the progression from a maxillary sinus infection to an external dental fistula, maxillary sinus abscess, and subcutaneous abscess.

Canine teeth are more likely to be involved in elderly patients because, compared to other teeth, the canine has a long and robust root, allowing it to be retained longer in life [14]. This increases the likelihood of caries affecting the canine. Additionally, the buccal area has been reported as the most common location for fistulas, occurring in 35.4% of cases [14]. In this case, she could not care for her mouth and teeth because of her ITP and steroid usage, causing her hemorrhagic diathesis. Organ-specific physicians need to focus on one organ, such as blood, heart, and lung. Thus, general physicians should care comprehensively for such patients to prevent complications.

General physicians should consider the possibility of atypical clinical courses when diagnosing external dental fistulas, particularly in elderly and immunocompromised patients [15]. External dental fistulas often present with few initial symptoms and are prone to misdiagnosis [2]. When a patient presents with facial swelling or a fistula, it is essential to consider dental diseases. Furthermore, early referral to an appropriate specialist is crucial, and a comprehensive diagnostic and treatment strategy can contribute to improved patient outcomes [16,17]. In patients with systemic complications, a holistic approach to management is essential, and preventive interventions to halt the progression of odontogenic infections should also be considered [18-20].

## Conclusions

This case underscores the complexities of managing external dental fistulas in immunocompromised elderly patients, where atypical presentations and rapid progression to severe complications, such as bone destruction and abscesses, can occur. Effective management requires early diagnosis, prompt referral to specialists, and a multidisciplinary approach, considering the patient’s broader health context, including systemic complications and immunosuppression. Maintaining oral hygiene is particularly challenging but essential in patients with conditions like ITP, where bleeding tendencies complicate routine care. This case highlights the need for comprehensive care strategies integrating dental and medical management, especially in patients with complex medical histories and polypharmacy. Early intervention and prevention are critical in improving outcomes and preventing further complications.
